# Timescale and genetic linkage explain the variable impact of defense systems on horizontal gene transfer

**DOI:** 10.1101/gr.279300.124

**Published:** 2025-02

**Authors:** Yang Liu, João Botelho, Jaime Iranzo

**Affiliations:** 1 Centro de Biotecnología y Genómica de Plantas, Universidad Politécnica de Madrid (UPM) - Instituto Nacional de Investigación y Tecnología Agraria y Alimentaria (INIA-CSIC), 28223, Madrid, Spain;; 2 Centro de Astrobiología (CAB), CSIC-INTA, 28850, Madrid, Spain;; 3 Institute for Biocomputation and Physics of Complex Systems (BIFI), University of Zaragoza, 50018, Zaragoza, Spain

## Abstract

Prokaryotes have evolved a wide repertoire of defense systems to prevent invasion by mobile genetic elements (MGEs). However, because MGEs are vehicles for the exchange of beneficial accessory genes, defense systems could consequently impede rapid adaptation in microbial populations. Here, we study how defense systems impact horizontal gene transfer (HGT) in the short term and long term. By combining comparative genomics and phylogeny-aware statistical methods, we quantify the association between the presence of seven widespread defense systems and the abundance of MGEs in the genomes of 196 bacterial and one archaeal species. We also calculate the differences in the rates of gene gain and loss between lineages that possess and lack each defense system. Our results show that the impact of defense systems on HGT is highly taxon and system dependent and, in most cases, not statistically significant. Timescale analysis reveals that defense systems must persist in a lineage for a relatively long time to exert an appreciable negative impact on HGT. In contrast, for shorter evolutionary timescales, frequent coacquisition of MGEs and defense systems results in a net positive association of the latter with HGT. Given the high turnover rates experienced by defense systems, we propose that the inhibitory effect of most defense systems on HGT is masked by their strong linkage with MGEs. These findings help explain the contradictory conclusions of previous research by pointing at mobility and within-host retention times as key factors that determine the impact of defense systems on genome plasticity.

Gene exchange plays a key role in the adaption of microbes to changing environments, facilitating the spread of antibiotic resistance, pathogenicity factors, metabolic genes, and other accessory functions (Arnold et al. 2022). Over the past decade, there has been an increasing interest in assessing the ecological and genetic factors that control horizontal gene transfer (HGT) and determine the outcome of newly acquired genes in microbial populations (Soucy et al. 2015; Hall et al. 2020; Lee et al. 2022). HGT is often mediated by mobile genetic elements (MGEs), such as phages, integrative and conjugative elements, and plasmids, against which bacteria have evolved an elaborate repertoire of defense systems (Doron et al. 2018; Botelho et al. 2023; Georjon and Bernheim 2023; Mayo-Muñoz et al. 2023; Shaw et al. 2023). As a result, large-scale patterns of HGT are shaped by an interplay of ecological and genetic variables that underlie cross-strain and cross-species differences in susceptibility to MGEs (Haudiquet et al. 2022).

Recent studies have highlighted the role of CRISPR-Cas as widespread adaptive immunity systems that protect archaea and bacteria against viruses and other MGEs (van Vliet et al. 2021; Watson et al. 2024). Although in vitro experiments have provided supportive evidence for this function (Marraffini and Sontheimer 2008; O'Hara et al. 2017; Watson et al. 2018), questions remain about the extent to which CRISPR-Cas systems constrain gene exchange in nature (Gophna et al. 2015; O'Meara and Nunney 2019; Shehreen et al. 2019; Westra and Levin 2020; Wheatley and MacLean 2021; Pursey et al. 2022). More generally, there is a paucity of research on how other defense systems, such as restriction-modification (RM), abortive infection (Abi), and an expansive repertoire of recently identified gene systems including Gabija, cyclic oligonucleotide-based antiphage signaling system (CBASS), DNA modification-based systems (DMSs), and defense-associated reverse transcriptases (DRTs) affect HGT in prokaryotes (Tesson et al. 2022; Costa et al. 2024).

In contrast with the expectation that defense systems restrict HGT by interfering with the propagation of MGEs, several empirical and theoretical observations suggest that the relation between defense systems and HGT might be more complex. First, the selection pressure to maintain defense systems in a population (and consequently their prevalence) generally increases with the exposure to MGEs (Oliveira et al. 2016; Meaden et al. 2022). Second, defense systems are mobilized by MGEs, which could lead to a trivial positive association with HGT rates. In a less trivial manner, whole defense systems or parts of them are often encoded by MGEs (Makarova et al. 2011; Pinilla-Redondo et al. 2022; Botelho 2023), promoting the retention of the latter for their beneficial side-effects in cellular defense (Koonin et al. 2020; Rocha and Bikard 2022).

Here, we investigated the association between seven widespread defense systems and HGT rates in 197 prokaryotic species. By combining high-quality genomic data, phylogenomic methods, and phylogeny-aware statistical inference, our study aimed to shed light on the nuanced consequences of the interplay between defense systems and MGEs on bacterial evolution across timescales.

## Results

### Association between defense systems and MGE abundance is MGE and taxon dependent

**Figure 1.**
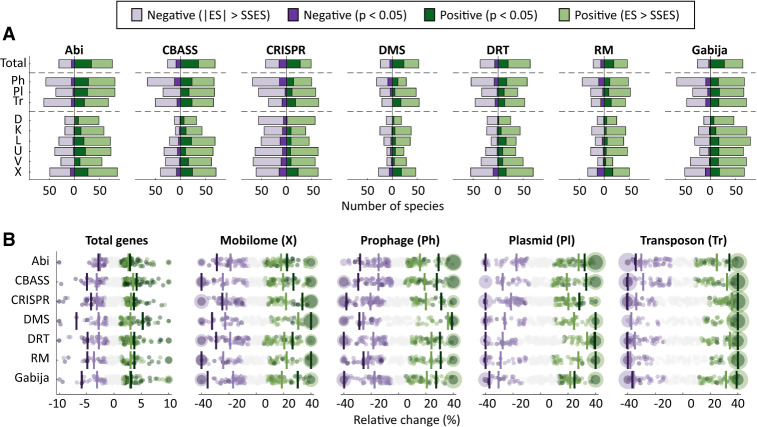
Association between seven widespread defense systems, total number of genes, and MGE abundance. (*A*) Number of species displaying positive or negative associations in a phylogenetic generalized linear mixed effect model (see Methods) according to two different criteria: statistical significance (*P* < 0.05) and absolute effect size greater than the smallest significant effect size (|ES| > SSES), separately computed for each response variable and defense system. (Note that because of cross-species differences in statistical power, the second criterion does not necessarily imply statistical significance.) The *top* row (total) indicates the association with the total number of genes. The association with MGEs was calculated based on marker genes for prophages (Ph), plasmids (Pl), and transposons (Tr). Abbreviations of functional categories: (X) mobilome; (L) replication, recombination, and repair; (U) intracellular trafficking and secretion; (K) transcription; (D) cell cycle control, cell division, and chromosome partitioning; and (V) defense. (*B*) Effect sizes, measured as relative differences in gene and MGE abundances. Each point corresponds to one species. Species with values beyond the axis limits are collapsed in a single point with size proportional to the number of species. Vertical lines indicate the median over all the species that show a positive or negative association according to the *P*-value and SSES criteria.

We used species-wise phylogenetic generalized linear mixed models (PGLMMs) to study the association between the presence or absence of the seven most-prevalent defense systems in the data set (RM, DMS, Abi, CRISPR-Cas, Gabija, DRT, and CBASS), genome size (measured as the total number of genes), and the number of MGEs per genome (Fig. 1A,B; Supplemental Tables S1–S4). Notably, the sign and magnitude of the associations are strongly taxon, system, and MGE dependent. Out of 197 species included in the analysis, around 20–30 (depending on the defense system) displayed a statistically significant (*P* < 0.05) positive association between the presence of the defense system and genome size. In contrast, statistically significant negative associations were only observed in five to 15 species (Fig. 1A, top row). Differences in the number of genomes per species could bias the assessment of statistical significance, leading to an overrepresentation of well-sampled taxa among those that display significant associations. Moreover, the detection of associations involving extremely abundant systems, such as RM and DMS (both with mean within-species prevalence close to 90%), could be compromised by insufficient statistical power. To overcome these limitations, we implemented a more permissive (but less biased) alternative criterion based on effect sizes to determine the number of positive and negative associations (see Methods). Regardless of the criterion, the presence of defense systems only correlates with genome size and MGE abundance in a minority of species. Overall, positive associations outnumber negative associations (2:1 and 1.5:1 positive-vs.-negative ratios for genome size and MGE abundance, according to the effect size criterion; 3.5:1 and 2:1 with statistical significance), although the trend is less pronounced in CRISPR-Cas systems (1.17:1 and 1:1 for genome size and MGE abundance; 1.75:1 and 1.5:1 with statistical significance). The analysis also reveals differences regarding the association between defense systems and distinct types of MGEs (Fig. 1A, middle rows). In all defense systems, negative associations with prophages are more frequent than were negative associations with plasmids (1.2–1.9 times more frequent, according to the effect size criterion; 1.8–4.7 with the statistical significance criterion). This is especially manifest in the case of CRISPR-Cas, whose presence correlates with a reduction in the number of prophages in 68 species (14 statistically significant) and with higher numbers of transposable elements and plasmids in 64 and 58 species, respectively (18 and 11 statistically significant). Significant associations, when detected, affect a sizeable fraction of the accessory genome, with 20%–40% differences in MGE content between genomes that do and do not harbor the defense system (Fig. 1B).

A more detailed analysis at the level of functional categories reveals that the presence of defense systems is most often associated with changes in the number of genes from COG categories mobilome (X); replication, recombination, and repair (L); intracellular trafficking and secretion (U); transcription (K); cell cycle control, cell division, and chromosome partitioning (D); and defense (V) (Fig. 1A; Supplemental Fig. S1). Genes from these functional categories are typically present in MGEs, suggesting that correlations (both positive and negative) between defense systems and genome size are primarily owing to differences in the abundance of MGEs. We confirmed that by masking genomic regions that correspond to known MGEs and rerunning the statistical analysis. As expected, 60% of the significant associations disappeared after masking MGEs (Supplemental Fig. S2). Although some significant associations persisted, a closer inspection revealed that those were often related to genes from degenerated prophages and other MGEs (such as phage satellites) that had not been originally identified as such and remained unmasked.

**Figure 2.**
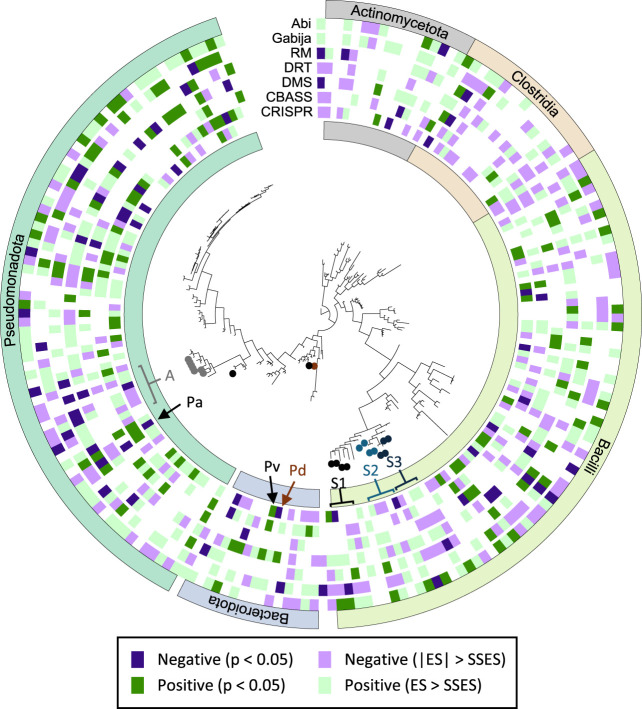
Taxonomic distribution of species displaying positive and negative associations between the presence of defense systems and the number of genes from the mobilome (based on COG annotations). Taxa discussed in the text are labeled: (A) *Acinetobacter*; (Pa) *Pseudomonas aeruginosa*; (Pv) *Phocaeicola vulgatus*; (Pd) *Phocaeicola dorei*; (S1) *Streptococcus pyogenes*, *Streptococcus dysgalactiae*, *Streptococcus equi*, and *Streptococcus uberis*; (S2) *Streptococcus gordonii*, *Streptococcus anginosus*, *Streptococcus mutans*, and *Streptococcus salivarius*; and (S3) *Streptococcus oralis*, *Streptococcus intermedius*, and *Streptococcus suis*. The classes *Bacilli* and *Clostridia* are the major components of the Genome Taxonomy Database phyla “Bacillota” and “Bacillota_A.” Note that, because of cross-species differences in statistical power, the condition |ES| > SSES does not necessarily imply statistical significance. For a larger version including all species names, see Supplemental Figure S3.

The sign of the association between defense systems and MGEs does not follow a clear taxonomic trend (Fig. 2; Supplemental Figs. S3, S4), with opposite signs sometimes found in closely related species (see, e.g., the differences for CRISPR-Cas in *Phocaeicola vulgatus* and *Phocaeicola dorei*). Moreover, the same species often display opposite trends for different defense systems. For example, in *Pseudomonas aeruginosa*, genomes with CRISPR-Cas contain fewer MGEs, whereas genomes with CBASS, Gabija, and RM systems are significantly enriched in MGEs. Negative associations between CRISPR-Cas and MGEs are more abundant in the genus *Acinetobacter* (five out of eight species), the phylum *Bacteroidota* (negative association in eight species, positive association in a single species), and the class *Clostridia*, the latter especially affecting prophages (negative association in 11 species, positive association in two species) (Supplemental Fig. S4). The genus *Acinetobacter* is also enriched in negative associations involving Abi (five species) and CBASS (four species). Furthermore, three almost-nonoverlapping groups of streptococci display negative associations for different defense systems. These encompass *Streptococcus pyogenes*, *Streptococcus gordonii*, *Streptococcus anginosus*, *Streptococcus mutans*, and *Streptococcus salivarius* in the case of CRISPR-Cas; *S. anginosus*, *Streptococcus oralis*, *Streptococcus intermedius*, and *Streptococcus suis* in the case of RM and DMS; and *Streptococcus pyogenes*, *Streptococcus dysgalactiae*, *Streptococcus equi*, and *Streptococcus uberis* in the case of Gabija.

The only archaeal species in the study (*Methanococcus maripaludis*) did not show any remarkable trend, other than a relatively strong (but not statistically significant) positive association between prophages and DMS/RM and between transposons and Gabija, as well as a moderate negative association between prophages and Gabija.

### Associations between defense systems and MGEs arise from differences in the rate of gene acquisition

**Figure 3.**
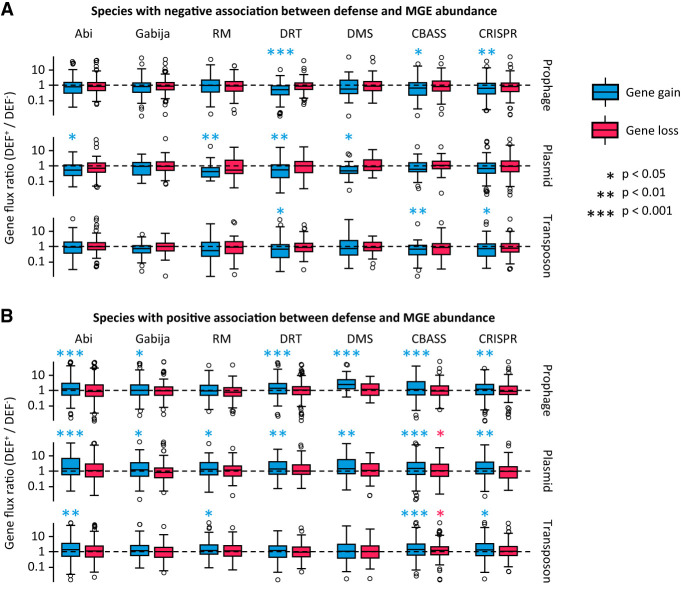
Relative differences in the rates of gene gain and loss between sister clades that do and do not harbor defense systems (DEF^+^ and DEF^−^, respectively). The boxplots represent the distribution of the DEF^+^/DEF^−^ ratio of gene-gain (or -loss) rates for each class of MGEs, calculated for every pair of sister clades. Values greater (or smaller) than one indicate increased (or reduced) gene flux in lineages that contain the defense system. (*A*) Species that show a negative association between the defense system and the number of marker genes for each class of MGEs (PGLMM with smallest significant effect size criterion). (*B*) Species that show a positive association between the defense system and the number of marker genes for each class of MGEs. In the boxplots, the *central* line indicates the median, the box limits correspond to the 25 and 75 percentiles, and the whiskers extend to the largest and smallest values not classified as outliers. *P*-values are based on a Wilcoxon test with log-ratio = 0 as null hypothesis.

To investigate if correlations between defense systems and MGEs involve cross-strain differences in genome plasticity, we built high-resolution strain trees and identified clades that contain a defense system (DEF^+^). Then, we inferred the rates of gene gain and loss associated with different classes of MGEs in DEF^+^ clades and in their respective sister groups lacking the defense system (DEF^−^) using the phylogenomic reconstruction tool GLOOME. We quantified the relative differences in gene gain and loss by dividing the rates observed in DEF^+^ clades by those from sister DEF^−^ clades. Finally, we compared the resulting DEF^+^/DEF^−^ gain and loss ratios between species in which MGE abundances and defense systems are positively and negatively correlated. We found that DEF^+^ and DEF^−^ clades often differ in their gain rates but not in their loss rates (Fig. 3A,B). Specifically, in species in which defense systems are associated with increased MGE abundance, gene-gain rates are typically higher in clades that contain the defense system. The opposite (i.e., a reduction of gene-gain rates in DEF^+^ clades) is observed in species in which defense systems are associated with lower MGE abundance. Among the latter, the biggest reductions in plasmid acquisition occur in association with RM, DRT, DMS, and Abi, whereas significant drops in prophage gain are observed for DRT, CBASS, and CRISPR-Cas. Taken together, these results confirm that correlations between defense systems and MGEs arise from cross-strain differences in gene gain rather than loss, as expected given the role of MGEs as facilitators of HGT.

### Timescales and linkage determine the sign of associations between defense systems and HGT

Because prokaryotic defense systems are often located within or adjacent to MGEs, we hypothesized that positive associations between defense systems and MGE abundance could be explained, at least in part, by recent cotransfer events (henceforth, we term this the “linkage hypothesis”). Under this hypothesis, positive associations would simply result from defense systems travelling together with MGEs.

**Figure 4.**
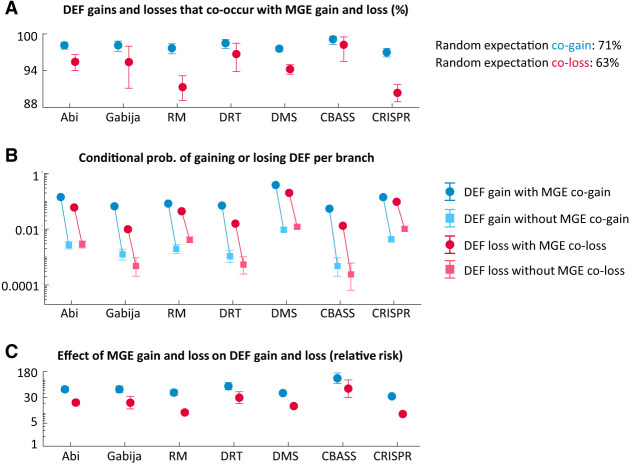
Co-occurrence of defense system gains and losses, and MGE gains and losses along the phylogeny. (*A*) Percentage of DEF gains (and losses) that occur in the same branch as an MGE gain (or loss). (*B*) Conditional probability of gaining (or losing) a defense system provided that an MGE is also gained (or lost) in the same branch compared with the conditional probabilities when an MGE is not acquired (or lost) in the same branch. (*C*) Effect of MGE gain (or loss) on the per branch probability to acquire (or loss) a defense system, measured as a risk ratio. Error bars in *A*,*B* correspond to 95% confidence intervals based on the binomial distribution. Error bars in *C* indicate the 95% confidence intervals for the relative risk (Morris and Gardner 1988).

To test the linkage hypothesis, we first verified that defense systems tend to be cotransferred with MGEs. We used ancestral reconstruction methods to identify the branches in which defense systems and MGEs were gained and lost along strain trees (Supplemental Table S5). We found that >95% of defense acquisition events occurred in branches in which MGEs were also gained, and >90% of defense losses occurred in branches in which MGEs were also lost (Fig. 4A). The random expectation given the rates of MGE gain and loss would be 71% and 63%, respectively (deviations with respect to these expectations are statistically significant with *P* < 10^−8^, binomial test). We also quantified the effect of MGE gain and loss on the per-branch probability of gaining or losing defense systems. The probability of acquiring a defense system is around 50-fold higher in branches in which MGEs are gained than in other branches (Fisher's exact test *P* < 10^−8^ for all defense systems) (Fig. 4B,C). Similarly, the probability of losing a defense system is 10-fold to 50-fold higher if MGEs are also lost in the same branch (Fisher's exact test *P* < 10^−20^ for all defense systems) (Fig. 4B,C). These trends are observed even in very short branches, spanning an evolutionary time of 10^−6^ substitutions per site in core genes (Supplemental Fig. S5). To rule out the possibility that these associations were owing to the presence of incomplete genomes, we separately considered terminal and internal tree branches. Because the data set only includes complete and nearly complete genomes, the absence of a small number of missing genes, if relevant, would only affect the inference of gene gain and loss in terminal branches (those leading from the immediate ancestor to each incomplete genome). Despite some quantitative differences, strong coacquisition (and co-loss) of MGEs and defense occurs in both terminal and internal branches (Supplemental Fig. S5), confirming that these associations are genuine.

Although co-gain of MGEs and defense systems along the same tree branch does not necessarily imply a single event of joint gain, the strength of the association, the extremely short timespans, and the fact that similar trends are observed for gene losses strongly suggest that concurrent gain and loss of MGEs and defense systems involve genetic linkage. (Alternative explanations in terms of strong selective pressure to quickly acquire defense mechanisms upon exposure to MGEs and lose them once the MGEs disappear might produce similar trends at the population level but not at the single-genome level, whereas episodic increases in the overall rate of HGT leading to separate but correlated acquisition of MGEs and defense systems would not explain correlated losses.)

**Figure 5.**
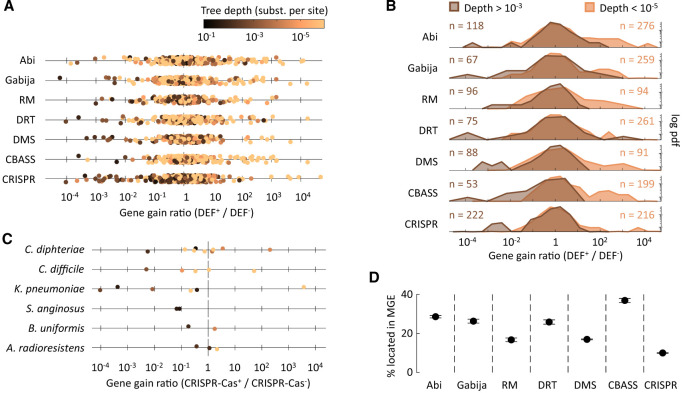
Association between defense systems and gene-gain rates depends on the timescale. (*A*) Ratio of overall gene-gain rates between sister clades that do and do not harbor defense systems (DEF^+^ and DEF^−^, respectively). Values greater (or smaller) than one indicate increased (or reduced) gene flux in lineages that contain the defense system. (*B*) Density distribution of the DEF^+^/DEF^−^ gain ratios, comparing clades that represent recent acquisitions of the defense system (depth < 10^−5^ substitutions per site in core genes) and clades that have retained the defense system for longer times (depth > 10^−3^ substitutions per site). The probability density function (*y*-axis) is represented in logarithmic scale to facilitate the visualization of the tails. The skewness of all distributions is statistically significant (Supplemental Table S7), with positive values for recent clades and negative values for deeper clades. (*C*) Representative examples of gene-gain ratios in shallow and deeper sister groups from the same species. (*D*) Percentage of genes from different defense systems located within known MGEs. Whiskers represent 95% confidence intervals based on the binomial distribution.

A major consequence of the cotransfer of MGEs and defense systems is that the negative effects of defense systems on HGT should be easier to detect at longer timescales or under evolutionary conditions that weakened genetic linkage. To test that prediction, we studied how relative differences in the rates of gene gain between DEF^+^ and DEF^−^ clades depend on the depth of their last common ancestor in the strain tree (note that the depth of the last common ancestor serves as an upper limit for the time that the defense system has been retained in a lineage). As expected, positive outliers (with much higher gene-gain rate in the DEF^+^ clade than in the DEF^−^ clade) almost invariably correspond to very recent lineages (fewer than 10^−5^ substitutions per base pair in nearly universal core genes), indicating a very recent acquisition of the defense system (Fig. 5A; Supplemental Table S6). In contrast, negative outliers (with much lower gene-gain rate in the DEF^+^ clade than in the DEF^−^ clade) generally correspond to deeper lineages (at least 0.001 substitutions per bp). We quantified these trends by calculating the skewness of the distribution of DEF^+^/DEF^−^ log-transformed gain ratios in very recent and older lineages (Fig. 5B; Supplemental Table S7). All the distributions show significant positive skewness in recent lineages and significant negative skewness in older lineages (all *P* < 0.001 except recent RM lineages, with *P* = 0.023; d'Agostino test for skewness). This quantitative analysis confirms that differences in timescale affect all defense systems, with positive associations between defense systems and gene gain being dominant in the short term and negative associations becoming more frequent in the long term.

A second prediction of the linkage hypothesis is that the net effect of defense systems on HGT is not only species specific but also lineage specific. That is, defense systems may display positive, zero, or negative association with HGT in different lineages depending on when the defense system was acquired and how tight is the linkage to MGEs. A more detailed study of gene-gain rates in individual species confirms that the effect of CRISPR-Cas is, indeed, lineage specific, with the same species encompassing recent CRISPR-Cas^+^ lineages that display increased gene-gain rates and older CRISPR-Cas^+^ lineages with reduced gene-gain rates (Fig. 5C). This observation, combined with the very recent acquisition of CRISPR-Cas in most lineages, could explain why many species show nonsignificant or positive associations between CRISPR-Cas and MGE abundances. Indeed, of the six representative species shown in Figure 5C, only *S. anginosus* and *Acinetobacter radioresistens* show a net negative association between CRISPR-Cas and MGE abundance in the PGLMM analysis.

The findings described so far indicate that, of the seven defense systems considered in this study, CRISPR-Cas is the one that most often induces a net reduction in HGT rates. According to the linkage hypothesis, this could be owing to a comparatively weaker physical association between CRISPR-Cas and MGEs. To evaluate that possibility, we calculated the percentage of genes from CRISPR-Cas located inside MGEs and compared that to other defense systems (Fig. 5D). Consistent with the linkage hypothesis, CRISPR-Cas is the defense system that is least often encoded by MGEs (10.0% vs. 20.1% for all other systems; *P* < 10^−10^, Fisher's exact test), followed by RM (16.7%) and DMS (17.0%).

### Anti-CRISPR proteins modulate associations between CRISPR-Cas and HGT

Anti-CRISPR proteins (Acr) have the potential to suppress the possible negative effect of CRISPR-Cas on MGE-driven gene transfer (Mahendra et al. 2020). This, in turn, could contribute to explaining the sign of the association between CRISPR-Cas and MGEs in different lineages. To assess that possibility, we searched all the genomes in the data set for known Acr, finding 16,058 proteins. Then, we compared the prevalence of Acr in species in which the presence of CRISPR-Cas is positively or negatively correlated with the MGE content, separately considering genomes that do and do not harbor CRISPR-Cas. Because Acr are generally encoded by MGEs (Pinilla-Redondo et al. 2020) and the prevalence of MGEs systematically varies across groups acting as a confounding factor, we restricted our comparisons to genomes that contain at least one prophage. Our results indicate that genomes with and without CRISPR-Cas differ in the prevalence of Acr (Supplemental Fig. S6). More importantly, these differences have opposite directions in species in which CRISPR-Cas is positively and negatively associated with MGE abundance. In the former, Acr are slightly more prevalent in genomes that contain CRISPR-Cas (46% vs. 43%, *P* = 0.026, Fisher's exact test). In the latter, Acr are less prevalent in genomes with CRISPR-Cas (35% vs. 41%, *P* < 10^−4^, Fisher's exact test). In both groups, the prevalence of Acr in genomes without CRISPR-Cas is similar. These results suggest that negative associations between CRISPR-Cas and HGT could be dependent on (or at least facilitated by) low prevalence of Acr in the genome.

## Discussion

Defense systems could have a significant impact on microbial evolution by effectively blocking the transfer of MGEs, reducing gene flow and limiting the spread of accessory genes. However, because defense systems are often carried by MGEs and these are the main vehicles of HGT, a net positive association between defense systems and gene exchange cannot be ruled out a priori. We assessed the relative weight of these two opposite scenarios by quantifying the association between defense systems, MGE abundance, and gene acquisition rates in a phylogeny-aware comparative study of 197 prokaryotic species.

Our results shed light on previous, apparently contradictory findings concerning the effect of CRISPR-Cas on genome evolution and diversification (Gophna et al. 2015; O'Meara and Nunney 2019; Shehreen et al. 2019; Wheatley and MacLean 2021; Pursey et al. 2022). A pioneering study conducted in 2015 found no evidence to support an overall association between CRISPR-Cas activity and reduced gene acquisition via HGT at evolutionary timescales (Gophna et al. 2015). Such lack of association was explained by several factors, including the high mobility of CRISPR-Cas systems, which limits their long-term impact on host genomes, and the possibility that HGT is mediated by MGEs that escape (or are not targeted by) CRISPR-Cas immunity.

Our analyses support the general conclusion that CRISPR-Cas and other defense systems have little overall impact on HGT in most bacterial species. Specifically, differences in the rates of gene acquisition in lineages that do and do not harbor defense systems are centered around zero. That said, we identified significant opposite trends at very short and intermediate evolutionary timescales: Positive associations between defense systems and HGT are more frequent at very short timescales, whereas negative associations become dominant at longer timescales. These opposite trends suggest that the possible negative effects of defense systems on gene exchange may be obscured by recent cotransfer events involving MGEs. Indeed, we found strong evidence of linkage between MGEs and defense systems, which tend to be not only coacquired but also co-lost from bacterial genomes at fast rates. Such joint dynamics of MGEs and defense systems manifest as a pattern of positive associations at short timescales. In turn, the negative effects of anti-MGE defense on HGT only become detectable if defense systems are maintained for long enough periods of time in the chromosome or in stable plasmids.

Besides this general picture, we identified some species in which the presence of defense systems (especially CRISPR-Cas) significantly correlates with smaller genome sizes and MGE abundances. Some of those associations had been previously described in *P. aeruginosa* and *Klebsiella pneumoniae* (Wheatley and MacLean 2021; Botelho et al. 2023). And yet, these species represent special cases rather than the rule, even in the context of host-associated bacteria. In fact, our results underline that the association between defense systems and HGT is strongly system and lineage dependent. This conclusion confirms and extends previous findings that showed that the impact of CRISPR-Cas on the spread of antibiotic resistance is highly variable across species and that its sign cannot be easily explained by simple ecological, environmental, or genomic variables (Shehreen et al. 2019).

The statistical modeling approach used in this study implies several assumptions and limitations that are worth discussing. First, phylogenetic corrections in PGLMM assume a linear association between the covariate (measured as nucleotide divergence in core genes) and the response variable (gene abundances). Therefore, the model can accommodate cross-strain variation in evolutionary rates as long as such variation also affects, in a correlated manner, the rates of gene gain and loss. Otherwise, heterogeneity in evolutionary rates would reduce the explanatory power of the phylogenetic term and the sensitivity of statistical tests. Second, variability in sample size, gene abundance, and prevalence of both defense systems and MGEs results in heterogeneous statistical power across species and systems. We introduced an effect size criterion to minimize possible biases, but one should still be cautious when interpreting some of the results. In particular, absolute numbers of positive and negative associations may be subject to sensitivity biases, whereas trends regarding the balance of positive and negative associations are likely more robust. Finally, we fitted all RM and DMS with one single model, even if the restriction sites and epigenetic modifications of these systems are very diverse. This could lead to an underestimation of effect sizes and statistical significance owing to the clumping of dissimilar trends.

Among the seven defense systems included in this study, CRISPR-Cas stands out for being more often associated with reduced genome sizes and lower MGE (especially prophage) abundances. In contrast, DMS, Abi, DRT, and CBASS are more often associated with higher numbers of MGEs and accessory genes. This finding is fully consistent with a recent study that compared 73 defense systems in 12 bacterial species (Kogay et al. 2024). We propose that what makes CRISPR-Cas systems different is their weaker (although still substantial) linkage with MGEs. Compared with fully functional CRISPR-Cas systems, other defense systems like Abi, Gabija, CBASS, DMS, and RM are more frequently located within or next to MGEs (Makarova et al. 2011; Benler et al. 2021; Rousset et al. 2022; Botelho 2023) and may have alternative functions related to MGE propagation. For example, Abi systems have been identified in phage-inducible chromosomal islands (PICIs) as accessory genes that facilitate their parasitic life cycle (Ibarra-Chávez et al. 2021). The narrow specificity of some defense systems may be another reason why those systems do not significantly interfere with HGT. For instance, the GmrSD type IV RM system selectively targets phages with glucosylated hydroxymethylcytosine (Bair and Black 2007), and the Thoeris defense system only appears to be effective against myoviruses (Doron et al. 2018). This caveat extends to any defense system based on epigenetic modifications, such as RM and DMS, whose overall effect on HGT depends on the repertoire of epigenetic markers in the host and MGE populations (Oliveira et al. 2016). Although selective targeting is often viewed as an outcome of phage–host coevolution, it is tempting to speculate that it could have been evolutionarily favored by the need to fight harmful genetic parasites while maintaining sufficiently high rates of HGT to prevent population-level gene loss (Iranzo et al. 2016).

Defense systems sometimes exhibit synergistic interactions (Dupuis et al. 2013; Wu et al. 2024), which could contribute to the heterogeneity of effects reported in this study. The vast number of potential interactions and the limited availability of high-quality genomes made it unfeasible to systematically account for the effect of interactions with the methods developed in this work. Determining if and to what extent synergy among defense systems affects HGT remains a subject for future investigation, possibly focused on a small set of experimentally validated interactions in highly sequenced species. Another open question concerns which levels of detail, both taxonomic and functional, best capture the effect of defense systems on HGT. From a taxonomic perspective, working at or below the species level is a natural choice because species represent genetically cohesive units (Bobay and Ochman 2017; Konstantinidis 2023; Conrad et al. 2024), and as a result, uncontrolled confounding factors are less likely to affect within-species than cross-species comparisons. In contrast, more complex multilevel approaches would be required to detect trends at higher taxonomic ranks. From a functional perspective, we grouped defense systems based on their mechanism of action, under the assumption that functionally similar systems produce similar effects on HGT. Although reasonable, this grouping criterion may not be optimal in systems in which subtypes markedly differ in their eco-evolutionary dynamics and linkage with MGEs. Moreover, fine-grain dissection of highly abundant systems, such as RM and DMS, could help improve the sensitivity of statistical tests by producing more balanced sets of strains with and without the subtypes of interest.

All in all, we showed that some defense systems, especially CRISPR-Cas, can significantly reduce HGT, although the effect is often masked by the fact that these systems travel together with MGEs. Beyond possible functional connections, the linkage between defense systems and MGEs is an inevitable consequence of the arms race between parasites and hosts. Because defense systems are costly and their efficacy drops as parasites evolve, they are subject to rapid turnover and depend on HGT for long-term persistence in microbial populations (van Houte et al. 2016; Iranzo et al. 2017; Koonin et al. 2017; Puigbò et al. 2017). As a result, it is extremely challenging to disentangle the impact of defense systems on gene flow from the causes that lead to their presence or absence, especially at short evolutionary timescales. As more and more genomic data become available, we expect that future research will overcome this challenge by quantifying the linkage between defense systems and MGEs, developing more realistic null models, and testing the role of defense systems on microbial adaptation at different timescales.

## Methods

### Genome collection and identification of defense systems

We parsed the Genome Taxonomy Database (GTDB; https://gtdb.ecogenomic.org) release 202 (Parks et al. 2020) to identify all high-quality genomes (according to the MIMAG criteria) (Bowers et al. 2017) with completeness >99%, contamination <1%, and contig count of fewer than 500. The 82,595 genomes that passed these filters were downloaded from the NCBI FTP site (https://ftp.ncbi.nlm.nih.gov). CRISPR-Cas systems were identified with CRISPRCasTyper v1.2.4 (Russel et al. 2020) using default parameters. We classified a genome as CRISPR-Cas^+^ if it contains at least one high-confidence Cas operon and CRISPR-Cas^−^ otherwise. Only the species with more than 10 genomes and at least five CRISPR-Cas^+^ genomes were further considered. To reduce the computational cost, we only considered a maximum of 500 genomes per species. Species with more than 500 genomes were randomly subsampled to keep at most 350 CRISPR-Cas^+^ and 150 CRISPR-Cas^−^ genomes. Other defense systems were identified with Padloc v1.1.0 (db v1.4.0) (Payne et al. 2022). Our analysis focused on the most prevalent defense systems: RM, DMS, Abi, CRISPR-Cas, Gabija, DRT, and CBASS.

After applying these criteria, 19,323 genomes belonging to 196 bacterial and 1 archaeal species (*sensu* GTDB) were included in the analysis (Supplemental Tables S1, S2). Of those, 2964 correspond to complete genomes and the rest to high-quality, nearly complete ones.

### Gene prediction and annotation

Open reading frames (ORFs) were predicted with Prodigal v2.6.3, using codon table 11 (prokaryotic genetic code) and “single” mode, as recommended for finished and draft quality genomes (Hyatt et al. 2010). Orthologous ORFs were then separately clustered for each species with Roary v3.13.0 (Page et al. 2015), setting an 80% identity threshold for initial clustering followed by synteny-based refinement (options “-t 11 -i 80”). The resulting gene clusters were functionally annotated by selecting a representative sequence, arbitrarily chosen among those with length between 0.95 and 1.05 times the average length of all sequences in the cluster. Representative sequences were functionally annotated by mapping them to in-house profiles of the Clusters of Orthologous Genes (COG) database (2020 release) (Galperin et al. 2021) with HMMER v3.1b2 (*e*-value < 0.001; http://hmmer.org). The 26 major prokaryotic functional categories defined in the COG database were assigned to the annotated genes. Some functional categories (A, RNA processing and modification; B, chromatin structure and dynamics; W, extracellular structures; T, signal transduction; and Z, cytoskeleton) were excluded because they rarely or never occur in prokaryotic genomes. The case-insensitive keywords “phage,” “plasmid,” and “transpos*” in the COG gene annotations were used to identify genes associated with prophages, plasmids, and transposons, respectively, and the resulting gene lists were manually curated to minimize false assignments. We based our statistical analyses on marker gene counts rather than full MGE counts because the latter are more susceptible to technical artifacts (e.g., different heuristics to deal with nested MGEs will affect the number of MGEs but not the number of MGE marker genes). Gene counts per genome and functional category are listed in Supplemental Table S8. Antidefense proteins, including anti-CRISPR, were identified by running HMMER v3.1b2 (*e*-value < 10^−10^) against the dbAPIS database (Yan et al. 2024).

### Identification of genomic regions containing MGEs

Prophages and plasmids were detected with geNomad v1.8.1 using default options (Camargo et al. 2024). Short transposons were identified based on the presence of isolated or paired genes annotated as transposases. In the latter case, we allowed for up to one additional gene between two transposon-related genes to account for the genetic architecture of some insertion sequences (Gómez et al. 2014). ICEfinder (Liu et al. 2019) was used to identify ICE and IME. The MGEs identified through these approaches were masked from complete genomes to produce the gene counts in Supplemental Table S9 and the results shown in Supplemental Figure S2.

### Species trees

Phylogenetic trees were separately built for each species based on the set of 120 prokaryotic marker genes (122 in the case of Archaea) proposed by the GTDB r202. For each species, only those marker genes with prevalence >80% were used for phylogenetic reconstruction. We aligned the amino acid sequences of each marker gene with mafft-linsi (L-INS-I algorithm, default options, MAFFT v7.475) (Katoh and Standley 2013) and back-translated the amino acid alignments to nucleotide alignments with pal2nal.pl v14 (Suyama et al. 2006) using codon table 11. After concatenating all nucleotide alignments, we built preliminary trees with FastTree v2.1.10 (options “-gtr -nt -gamma -nosupport -mlacc 2 –slownni”) (Price et al. 2010). The tree topologies produced by FastTree were subsequently provided to RAxML v8.2.12 (Stamatakis 2014) for branch length optimization (raxmlHPC with options “-f e -m GTRGAMMA”). The final trees are included in Supplemental File S1.

To visualize trends across species (Fig. 2; Supplemental Figs. S3, S4), we used the online tool iTOL (Letunic and Bork 2021) and the multispecies tree from GTDB r202.

### Phylogenetic generalized linear mixed models

For each genome in the data set, we collected the following response variables: the total number of genes, the number of genes belonging to each functional category, and the number of genes associated with prophages, plasmids, and transposons (Supplemental Table S8). Genes that belong to the seven defense systems of interest were excluded when computing these values. Then, for each response variable, we fitted a PGLMM with Poisson distribution and canonical link function, using the presence or absence of each defense system as predictors and the species trees as guides to generate the covariance matrix.

For each species, the PGLMM assumes that the response variable, *Y*_*i*_, follows a Poisson distribution with mean *μ*_*i*_, that is, $Y_{i}∼\mathrm{Poisson}(\mu_{i})$. The expected gene abundance, *μ*_*i*_, is modeled as $\log\mu_{i}={\text{β}}_{0}+\sum {\text{β}}_{j}X_{ij}+\epsilon_{i}$, where *β*_0_ is the intercept, *β*_*j*_ is the coefficient associated with the defense system *j*, and *X*_*ij*_ ∈ {0, 1} denotes the absence or presence of defense system *j* in genome *i*. The random effects $\epsilon_{i}$ follow a multivariate normal distribution, $\epsilon ∼\mathrm{Gaussian}(0,\sigma_{\;phy}^{2}C)$, where $\sigma_{\;phy}^{2}$ is the strength of the phylogenetic signal, and C is a covariance matrix derived from the phylogenetic tree under the assumption of Brownian motion evolution.

To extend the PGLMM to multiple species, we considered that the effect of defense systems on gene content may not be the same across species (this was a reasonable assumption a priori and later confirmed by the analysis). To account for that, the multispecies model must include an interaction term “defense × species,” whose coefficients are relevant per se and, accordingly, modeled as fixed effects. Moreover, because HGT rates are highly variable among species (Puigbò et al. 2014; Iranzo et al. 2019), it would be inadequate to extrapolate phylogenetic corrections beyond single species or assume that the strength of the phylogenetic signal is the same for all species. These considerations are captured by a phylogenetic covariance matrix with block-diagonal structure (one block per species) and species-wise values of the phylogenetic coefficient (one for each block of the phylogenetic covariance matrix). In practice, fitting a multispecies model with these specifications is formally equivalent to fitting independent models for each species. The latter approach, that we adopted, has the advantage of being more suitable for parallelization and requiring fewer computational resources. Thus, for each species and response variable, we fitted a PGLMM with the function pglmm_compare(response_variable ∼ Abi + CBASS + CRISPR + DMS + DRT + Gabija + RM, family = “poisson,” data = SpData, phy = SpTree) from the R package phyr v1.1.0 (Li et al. 2020). In the formula, Abi, CBASS, CRISPR, and so on are binary variables representing the presence (one) or absence (zero) of each defense system. Supplemental Table S4 presents the coefficients, *P*-values, and goodness of fit of the model.

The model described above includes nine coefficients (intercept, seven defense systems, and the phylogenetic signal), which could lead to overfitting in species in which the number of available genomes is limited. As an alternative, we also fitted seven separate PGLMM, one for each defense system, involving a single predictor and the phylogenetic random effect (Supplemental Table S3). This approach does not account for correlations among defense systems, but it has the advantage of not being affected by overfitting. The figures in the article are based on this second set of models, although, in practice, both approaches produce very similar quantitative results.

For each class of PGLMM, we also fitted nonphylogenetic models in which the covariance matrix C of the random effect was replaced by the identity matrix. The PGLMM were compared to their nonphylogenetic counterparts using the conditional Akaike information criterion (cAIC) as previously described (Greven and Kneib 2010; Säfken et al. 2021). Based on the cAIC, PGLMM performed better than nonphylogenetic models in 65% of the species-response-defense triplets and were close to nonphylogenetic models (ΔcAIC < 2) in another 25% of the triplets (in most of those cases, the strength of the phylogenetic signal was close to zero, which made both phylogenetic and nonphylogenetic models equivalent).

To account for the possibility that other (less abundant) defense systems could explain part of the variability in the results, we explored a more complex set of models that included the total number of other defense systems as an additional predictor. These models generally performed worse than their simpler variants (ΔcAIC > 0 in 83% of the species) and were not considered for further analysis.

Large differences in sample size among species and defense systems translate into unequal precision in the estimation of the model coefficients. To deal with that limitation, we used two different criteria to identify species in which the presence of a defense system is positively or negatively associated with gene numbers. For one option, we adopted a classical criterion of statistical significance (*P* < 0.05) for the predictor variable in the PGLMM. For the other option, we applied an alternative criterion based on effect size, aimed at comparing species with different sample sizes in which *P*-values are not commensurable. Specifically, for each variable and defense system, we jointly considered the PGLMM of all the species and determined the smallest effect size (in absolute value) that reached statistical significance (*P* < 0.05) in any species. Then we used the smallest significant effect size (SSES) as a threshold to classify associations as positive, negative, or null.

### Inference of gene gain and loss

Gene gains and losses at each branch of each species tree were estimated with GLOOME (Cohen and Pupko 2010), using as inputs the gene presence/absence matrices previously generated by Roary and the species trees. The parameter configuration file was set to optimize the likelihood of the observed phyletic profiles under a genome evolution model with four categories of gamma-distributed gain and loss rates and stationary frequencies at the root.

### Comparison of gene-gain and gene-loss rates between DEF^+^ and DEF^−^ clades

For each species tree, we defined DEF^+^ clades as the narrowest possible clades such that at least 80% of the leaves contain the defense system of interest. Candidate DEF^−^ clades were defined in an analogous way, referring to leaves without the defense system. Next, we identified pairs of DEF^+^ and DEF^−^ clades that constitute sister groups. Sister DEF^+/−^ pairs were excluded if both clades contained a single genome. For each clade in a valid pair, we computed the overall gene-gain and gene-loss rates as the expected number of gene gains (or losses) in that clade divided by the total branch length. We calculated gain and loss rates for different functional categories and MGEs in an analogous way but restricting the sum of gene gains and losses to the genes of interest. When calculating overall and category-wise gain and loss rates, we did not take into account the contribution of species-wise singletons (genes without homologs in other genomes of the same species), as they may represent false gene predictions or genes that are replaced at unusually high rates (Wolf et al. 2017). To account for the non-negative nature of gain and loss rates and their heavy-tailed distributions, comparisons between DEF^+^ and DEF^−^ sister branches were done based on log-transformed rate estimates. To calculate the skewness of the distributions and their statistical significance, we use the method proposed by D'Agostino et al. (1990) as implemented by the scipy.stats.skewtest() function in Python (Virtanen et al. 2020).

## Supplemental Material

Supplement 1

Supplement 2

Supplement 3

Supplement 4

Supplement 5

Supplement 6

Supplement 7

Supplement 8

Supplement 9

Supplement 10

Supplement 11

Supplement 12

Supplement 13

Supplement 14

Supplement 15

Supplement 16
