# Supplementary material for: Timescale and genetic linkage explain the variable impact of defense systems on horizontal gene transfer
Source: Genome Res. 2025 Feb;35(2):268–78. doi: 10.1101/gr.279300.124 (PMC11874982; doi:10.1101/gr.279300.124)
Supplement: Supplement 3 [file Supplemental_Fig_S3.pdf]

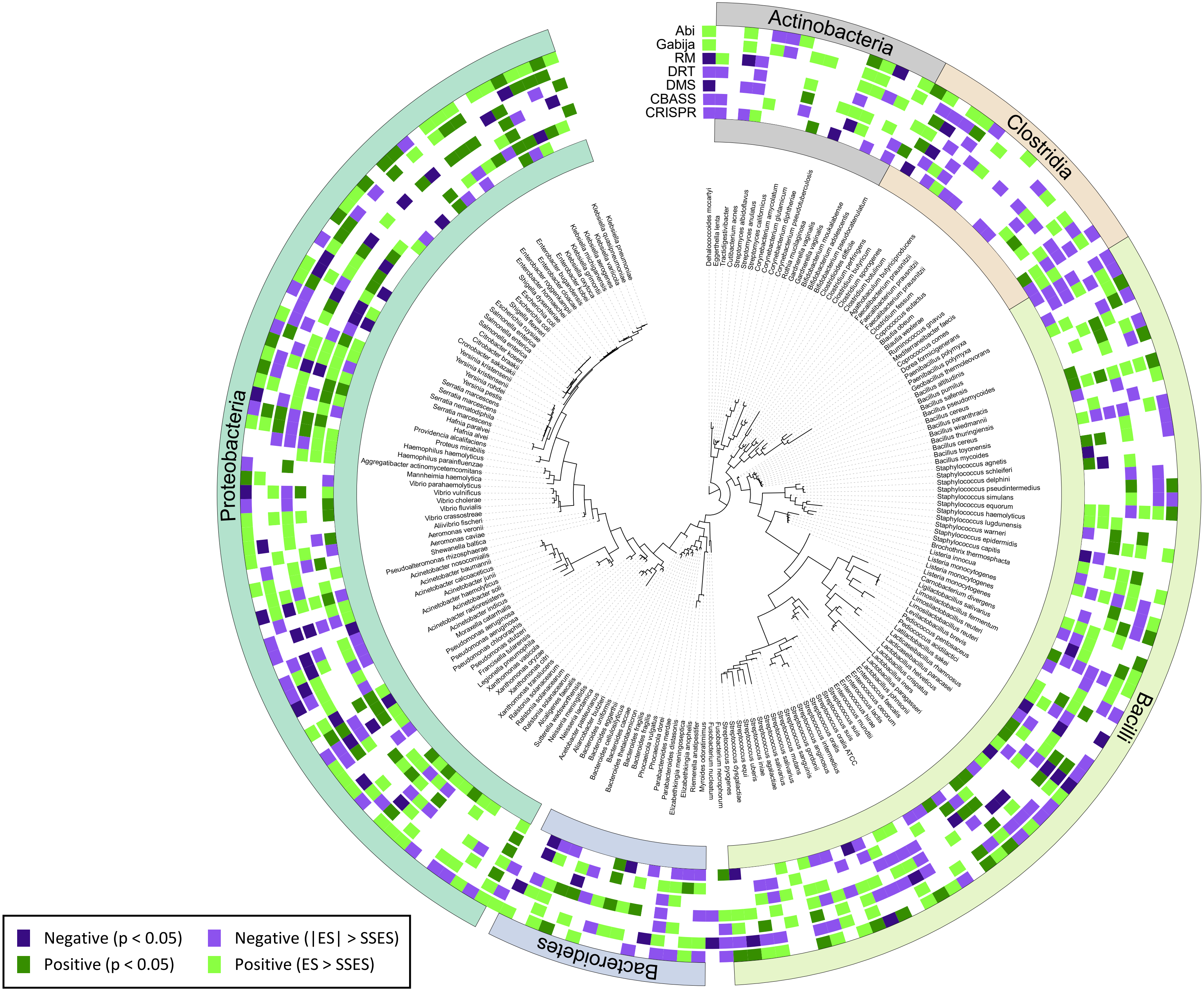

Figure S3: Taxonomic distribution of species displaying positive or negative associations between the presence of defense systems and the number of genes from the mobilome (based on COG annotations).
